# Advances in nanomaterial-based delivery systems for inducing transplantation tolerance

**DOI:** 10.3389/fimmu.2026.1908621

**Published:** 2026-08-26

**Authors:** Qingqing Li, Yun Zhang, Jinwei Wu, Dongxing Huang, Zhongquan Qi

**Affiliations:** 1 Guangxi Key Laboratory of Special Biomedicine, School of Medicine, Guangxi University, Nanning, China; 2 Infectious Disease Laboratory, Guangxi AIDs Clinical Treatment Center, The Fourth People’s Hospital of Nanning, Nanning, Guangxi, China; 3 Fujian Duanneng Cell Technology Co., Ltd., Xiamen, Fujian, China

**Keywords:** delivery systems, immune regulation, nanomaterials, organ transplantation, transplantation tolerance

## Abstract

Organ transplantation is the primary therapeutic approach for patients with end-stage organ failure. However, challenges such as transplant rejection, the toxic side effects of long-term systemic immunosuppression, and substantial economic burdens remain pressing issues in clinical practice. The goal of inducing donor-specific transplantation tolerance is considered the most effective strategy to address these problems. Conventional tolerance-inducing strategies, including hematopoietic chimerism establishment, costimulatory signal blockade, and regulatory cell therapy, are often hampered by key limitations such as myeloablative toxicity, resistance from memory T cells, high costs associated with *ex vivo* cell expansion, and poor *in vivo* stability. This review summarizes the types and properties of nanomaterials used to induce transplantation tolerance, systematically discusses their payload categories and immunoregulatory mechanisms, and delineates key delivery strategies and *in vivo* mechanisms. Furthermore, it analyzes current challenges and bottlenecks faced by nanodelivery systems. Finally, future perspectives on optimizing and translating these systems into clinical applications are proposed, providing valuable insights for developing safe, precise, and efficient strategies for transplantation tolerance induction.

## 1 Introduction

Organ transplantation is the first-line clinical treatment for patients with end-stage organ failure (1, 2). Recognition of human leukocyte antigen (HLA) mismatches by adaptive immune cells constitutes the core mechanism triggering transplant rejection (3, 4), which further mediates the occurrence of T cell-mediated or antibody-dependent rejection (5–7). To suppress immune rejection, patients require lifelong medication, which not only induces toxic side effects but also imposes a substantial economic burden. Therefore, inducing allograft tolerance has long remained the ultimate goal in organ transplantation (8, 9).

Current strategies for transplantation tolerance induction are primarily categorized into central and peripheral immune tolerance (10). Central immune tolerance induction is centered on the establishment of hematopoietic chimerism (11–13). This approach typically involves radiation, chemotherapeutic agents, or antibody-mediated T cell depletion, combined with hematopoietic stem cell transplantation, to achieve immune system reconstitution in the recipient, ultimately culminating in hematopoietic chimerism (14, 15). However, it is prone to inducing graft-versus-host disease (GVHD), which severely hinders its clinical translation. Subsequent studies have implemented immune reconstitution through CD4 antibody-mediated T-cell depletion (16) or myeloablative irradiation (17), combined with retroviral vector-mediated transfection of donor major histocompatibility complex (MHC) class I or II genes and bone marrow cell infusion. Although this can circumvent GVHD, ethical and safety controversies remain. Furthermore, due to thymic involution in adults, particularly the elderly, this strategy is prone to severe complications such as impaired immune reconstitution (18).

Strategies for peripheral immune tolerance induction primarily encompass costimulatory signal blockade and regulatory cell therapy (19). Cytotoxic T lymphocyte-associated antigen 4-immunoglobulin (CTLA4-Ig) fusion protein is the only costimulatory blockade strategy that has advanced into clinical studies. It blocks the interaction between CD80/CD86 on antigen-presenting cells and CD28 on T cells, thereby impairing T-cell activation. When T cells receive T cell receptor signaling in the absence of CD28-mediated costimulation, this blockade triggers T-cell apoptosis, anergy, or differentiation into regulatory T cells (Tregs), ultimately mediating immune tolerance (20, 21). Nevertheless, this strategy is susceptible to resistance by memory T cells, resulting in suboptimal tolerance induction.

Regulatory cell therapy achieves tolerance induction by *ex vivo* induction, expansion, and adoptive transfer of donor-specific regulatory cells, including Tregs (22–24), regulatory B cells (Bregs) (25), and regulatory dendritic cells (26). Its core mechanisms involve low expression of costimulatory molecules such as CD80, CD86 and CD40, and secretion of inhibitory cytokines including interleukin-10 (IL-10) and transforming growth factor-β (TGF-β), enabling precise modulation of donor-specific T cells (27, 28). Although relevant clinical trials have been initiated in multiple centers, clinical translation remains restricted by challenges such as inconsistent quality and quantity of source cells, high costs of *ex vivo* expansion, inadequate quality control systems, and difficulty maintaining *in vivo* stability (29). Furthermore, some immunosuppressive drugs and therapeutic regimens can induce regulatory cell generation *in situ* within recipients, thereby actively inducing transplantation tolerance (30, 31). But these strategies face limitations including insufficient targeting and adverse systemic effects associated with systemic immunosuppression.

However, both T-cell depletion and costimulatory signal blockade strategies encounter common barriers in clinical translation. Although hematopoietic chimerism induction can establish central immune tolerance, the toxicity of myeloablative conditioning is severe, predisposing recipients to GVHD and impaired immune reconstitution. Costimulatory blockade strategies target core pathways including CD28-B7 and CD40-CD40L to block the second signal required for T-cell activation, yet memory T cells bypass CD28-dependent activation via alternative costimulatory axes (OX40, 4-1BB, LFA-1) and IL-15/CD122 signaling to trigger alloimmunity, thereby resulting in suboptimal tolerance induction. Regulatory cell therapy exhibits precise targeting, yet its clinical advancement is restricted by high costs of *ex vivo* expansion and poor *in vivo* stability.

Nanomaterial-based delivery systems have recently entered the field of transplantation tolerance research (32, 33). First, nanomaterials possess favorable biocompatibility and biodegradability, which reduce host damage and improve the safety profile for clinical application. Second, advances in nanotechnology enable the delivery of immunoregulatory molecules using both cell-derived and non-cell-derived biomaterials, facilitating specific targeting of alloantigens within the recipient (34, 35). Third, nanomaterials permit the co-delivery of donor antigens and immunosuppressive drugs with reduced sensitivity to the immune microenvironment, holding promise for more precise and stable induction of donor-specific immune tolerance *in vivo* (36).

This review offers a comparative classification of four major nanocarrier platforms against unified criteria including loading efficiency, targeting capability, manufacturing scalability, and translational readiness. We also provide a mechanistic framework that connects nanocarrier properties and delivery strategies to their *in vivo* tolerogenic outcomes, organized around three core delivery modalities: tolerance inducing organ targeting, immune cell targeting, and local graft site delivery. In addition, we analyze translational barriers across manufacturing, regulatory, and biological domains and propose priority areas for future research to bridge the gap between preclinical findings and clinical application. Structurally, we begin with a comparative analysis of nanocarrier platforms (Section 2), followed by a systematic categorization of five payload types and their immunomodulatory mechanisms (Section 3). We then delineate three delivery strategies and their *in vivo* mechanisms (Section 4), analyze translational challenges (Section 5), and conclude with a summary of the current landscape and future directions (Section 6). Collectively, this review bridges materials science, immunology, and clinical translation, offering a roadmap for nanocarrier design beyond isolated platform descriptions.

## 2 Types, properties, applications and comparative analysis of nanodelivery materials

### 2.1 Synthetic polymeric nanomaterials

Polylactic-co-glycolic acid (PLGA) are the most widely used synthetic polymeric nanomaterials for fabricating targeted delivery nanoparticles. It typically ranges from 100 to 250 nm and are negatively charged. PEGylation or chitosan coating can shift this charge to neutral or positive (37). For instance, Shahzad et al. (38) used PLGA carriers to deliver nucleic acids, proteins, peptides, or drugs into target tissues with sustained release kinetics. Surface conjugation of CD47-Fc reduced uptake by the mononuclear phagocyte system. This formulation prolonged skin allograft survival in a donor-specific manner by eliminating H-2K^b^-specific alloreactive CD8^+^ T cells through Fas-mediated apoptosis and proliferative inhibition driven by programmed death ligand 1 (PD-L1) and TGF-β. However, this study was validated only in a single-MHC-mismatched murine model with weak immunogenicity, placing it at the proof-of-concept stage with limited translational relevance (39).

Shah et al. (40) and Bryant et al. (41) utilized PLGA nanoparticles for efficient delivery of donor antigens. Donor-derived MHC molecules represents the major alloantigens. After PLGA nanoparticles are internalized by splenic macrophages and dendritic cells (DCs) of the mononuclear phagocyte system, encapsulated donor antigens undergo intracellular proteolytic processing and peptide loading onto recipient MHC complexes to mediate indirect allorecognition. These processed antigenic epitopes further initiate anti-donor immune responses and activate recipient T cells (42). If the selected donor MHC peptides cover all mismatched MHC loci between donor and recipient, donor-specific immune tolerance may potentially be induced in the recipient, offering a strategy for establishing transplantation tolerance. Nevertheless, both studies were validated only in weakly immunogenic murine models with partial efficacy, placing them at the proof-of-concept stage.

Further studies have shown that PEGylation of PLGA can improve nanoparticle pharmacokinetics and reduce protein adsorption, which could facilitate the delivery of immunomodulatory agents to target tissues in transplantation settings (43, 44). PEGylated PLGA suppress complement cascade activation, hinder neutrophil uptake and recruitment, and downregulate surface expression of CD80/CD86 on local DCs at injection sites (45–49). These effects collectively facilitate the formation of an *in vivo* tolerogenic immune microenvironment conducive to antigen-specific allogeneic tolerance.

### 2.2 Natural polymeric nanomaterials

In addition to synthetic polymers, natural polymers are also widely utilized for drug delivery systems. Natural polymers include cationic chitosan and polylysine, neutral cellulose and dextran, and anionic hyaluronic acid. The intrinsic positive charge of cationic nanoparticles facilitates their adhesion to negatively charged cell membranes, a property that markedly enhances cellular uptake of these nanoparticles (50, 51).

For instance, rapamycin (RAPA) delivered via chitosan/polylactic acid nanoparticles achieves enhanced intraocular drug accumulation through electrostatic interactions, suppressing corneal transplant rejection and prolonging graft survival compared with free RAPA. However, validated only in an immune-privileged rabbit corneal model, this local delivery strategy has limited relevance to systemic transplantation tolerance (52, 53). Furthermore, alkylated hyaluronic acid hydrogels loaded with Treg-derived extracellular vesicles (EVs) engineered to express forkhead box P3 (FOXP3) have been developed for immunomodulation in kidney transplantation. This strategy promotes local enrichment of FOXP3^+^ Tregs, which secrete anti-inflammatory cytokines such as IL-10 and TGF-β, and upregulates PD-L1 expression on local antigen-presenting cells (APCs). These concerted actions suppress CD8^+^ T cells and T helper 1 (Th1) responses, reduce M1 macrophages and promote M2 polarization, alleviate graft inflammation, diminish complement C3/IgG deposition, and ultimately prolong allograft survival with reduced chronic fibrosis. This strategy is among the most promising in mechanistic validation and translational exploration, with preliminary feasibility confirmed in a porcine model. Nonetheless, the three-step manufacturing process is complex and survival remains prolonged rather than indefinite, with long-term safety unconfirmed (54).

### 2.3 Lipid-based nanomaterials

Lipid nanoparticles are classified into four main types: solid lipid nanoparticles, nanostructured lipid carriers, liposomes, and polymer-lipid hybrid nanoparticles. Solid lipid nanoparticles consist of solid lipids with surfactants. Particle size and surface charge vary with composition and pH. Nanostructured lipid carriers blend solid and liquid lipids to form a disordered matrix with improved loading capacity. Liposomes are phospholipid vesicles ranging from 30 nm to several micrometers, with a bilayer thickness of approximately 4–5 nm. Polymer-lipid hybrid nanoparticles combine polymers and lipids for enhanced stability and biocompatibility (55).

Compared with polymers, lipids possess inherent advantages as nanodelivery materials. They are natural components of cell membranes and thus less likely to induce inflammatory responses. Moreover, the intrinsic bilayer architecture of lipids facilitates the co-delivery of both hydrophilic and hydrophobic payloads. Furthermore, lipids can undergo diverse modifications, rendering them efficient and targeted drug delivery carriers (56). Antigen-encoding messenger RNA (mRNA) delivery via lipid nanoparticles, which has played a key role in COVID-19 mRNA vaccine development (57–59) and has been extensively investigated in cancer therapy (60–63), and may hold promise for future application in transplantation tolerance (64).

### 2.4 EVs

EVs, which mainly comprise exosomes (40–120 nm), microvesicles (50–1000 nm), and apoptotic bodies (500–2000 nm), are secreted by nearly all cell types including immune cells and play a critical role in intercellular communication (65). Recent studies have shown that EVs exert dual regulatory effects on alloimmune responses, capable of either driving allograft rejection or promoting transplant tolerance depending on their cellular origin and molecular cargo (66, 67).

Among these, exosomes derived from immature DCs (imDex) have drawn particular attention in transplantation tolerance. Although imDCs themselves are limited by poor *in vivo* survival and instability, their exosomal counterparts imDex retain the immunomodulatory features of parental cells while overcoming these drawbacks. Donor-derived imDex combined with short-term LF15–0195 induced donor-specific tolerance in a rat cardiac model, as evidenced by indefinite graft survival, acceptance of donor-matched secondary grafts and rejection of third-party allografts, and adoptive transfer of tolerance. The combination inhibited donor-specific T-cell proliferation via both direct and indirect allorecognition pathways, with regulatory activity mediated by non-T splenocyte populations. The reliance on LF15–0195 and labor-intensive exosome preparation limits the translational feasibility of this otherwise promising approach (68).

Similarly, Treg-derived exosomes prolonged graft survival through a different mechanism. They arrest in CD8^+^ cytotoxic T lymphocytes at the G0/G1 phase of the cell cycle, suppressing T cell proliferation with reduced interferon-gamma (IFN-γ) and perforin. This effect was reversed by GW4869, an inhibitor of exosome biogenesis, confirming exosome-mediated suppression. Donor-type Treg exosomes outperformed autologous ones, whereas B-cell exosomes promoted proliferation, highlighting the donor-specific and cell-origin-dependent nature of this effect. However, Treg-derived exosomes face inherent bottlenecks including low yield, complex manufacturing, and delayed rejection rather than true tolerance induction (69, 70).

### 2.5 Novel nanomaterials

In addition to the aforementioned nanomaterials, hybrid nanodelivery systems are fabricated by integrating synthetic nanoparticles with cell-derived components. These carriers were originally developed and rapidly progressed for cancer therapy (71–74). However, their application for inducing antigen-specific tolerance in transplantation remains scarce (75). Moreover, enzyme-responsive nanocarriers constitute an emerging class of delivery platforms. Luo et al. (76) constructed tacrolimus prodrug-based enzyme-responsive nanoparticles that enable selective accumulation of immunosuppressants within grafts through MMP9-triggered micelle-to-aggregate morphological transition, thereby suppressing T-cell infiltration and proliferation, increasing the intragraft Treg/effector T-cell ratio, reducing hepatocyte apoptosis and oxidative damage, improving liver function parameters, and ultimately prolonging allograft survival with accelerated body weight recovery in a rat liver transplantation. At the proof-of-concept level, these materials indeed demonstrate unique advantages in stimuli-responsive delivery, yet their translation to tolerance-inducing applications remains to be established.

### 2.6 Physicochemical properties of nanocarriers

Particle size is a key determinant of nanoparticle immune recognition and the resulting immune response. Particles smaller than 100 nm are preferentially taken up by DCs and drain to lymph nodes, whereas those larger than 500 nm are cleared by macrophages (77). Size additionally affects complement activation, which diminishes as particle size increases. Particles below 50 nm enter via receptor-mediated endocytosis, whereas larger particles more readily activate inflammasomes (77, 78).

Surface charge dictates the initial interaction between nanoparticles and cell membranes and subsequent immunological recognition. Positively charged nanoparticles are easily internalized by APCs and activate pro-inflammatory pathways, exerting adjuvant effects, yet may also induce cytotoxicity and non-specific inflammation. In contrast, negatively charged nanoparticles exhibit lower uptake efficiency but better biocompatibility. Surface charge also influences complement activation pathways, with negative charges primarily activating the classical pathway and positive charges favoring the alternative pathway (78, 79).

Morphology influences cellular uptake, and circulation. Spherical nanoparticles facilitate uptake by APCs due to their high symmetry, yet are readily cleared by macrophages. Non-spherical nanoparticles, such as rods and disks, exhibit reduced phagocytic clearance and prolonged circulation owing to their higher aspect ratios (77, 78).

Surface functionalization plays a critical role in modulating nanoparticle targeting and immune recognition. Targeting ligands such as mannose or ApoB peptide can direct nanoparticles to specific hepatic cell subsets, although their function may be compromised by protein corona shielding. PEGylation reduces protein adsorption and non-specific immune recognition to prolong circulation, but PEG itself may induce anti-PEG antibodies and accelerated clearance upon repeated administration. Biomimetic surface decoration via CD47, FasL or PD-L1 moieties imparts nanoparticles with self-recognition signals or direct tolerogenic activity, yet they carry the risk of introducing novel immunogenic epitopes (79).

Upon entering biological fluids, nanoparticles are rapidly coated with proteins, forming a protein corona. This dynamic layer alters their effective size and surface charge, which in turn affects colloidal stability and cellular uptake. Meanwhile, the protein corona modulates immune recognition through its specific protein composition, influencing circulation time, biodistribution, and targeting efficiency. However, corona formation may also shield targeting ligands or introduce new immunogenic epitopes (80).

After endocytic uptake, nanoparticles are delivered to endosomes and lysosomes, where acidic pH and degradative enzymes promote polymer breakdown. For PLGA and related polymeric nanoparticles, degradation occurs via hydrolytic cleavage of ester bonds. The rate of degradation depends on polymer composition, molecular weight, and size. Smaller particles tend to hydrolyze faster (81).

The high surface area and short diffusion path of nanoparticles complicate drug release control compared with conventional micron-sized carriers. Release behavior is largely determined by two factors: diffusion through the polymer matrix and degradation of the carrier itself. In diffusion-controlled systems, drug transport depends on movement through the polymer matrix. In degradation-controlled systems, release relies on hydrolytic breakdown of polyester backbones such as PLGA and PLA, with rates influenced by molecular weight, end-group chemistry, and crystallinity. Stimuli-responsive carriers are designed to trigger release in response to pH, enzymatic activity, or redox potential (82).

### 2.7 Comparative analysis of nanocarrier platforms

Loading efficiency and targeting capability are two key parameters for assessing nanocarrier platforms. Lipid nanoparticles generally achieve higher encapsulation efficiency than polymeric nanoparticles, especially for nucleic acid cargoes. Polymeric nanoparticles offer tunable release kinetics and good biocompatibility, but their loading capacity is comparatively limited. Lipid-based systems also support dual-compartment loading, allowing simultaneous encapsulation of both hydrophilic and hydrophobic agents. EVs display substantially lower loading efficiency than either synthetic platform, owing to their restricted internal volume and the lack of robust active loading strategies (83–85).

In terms of targeting, polymeric and lipid nanoparticles typically require exogenous surface functionalization to achieve cell-specific delivery. However, the intrinsic homing properties of EVs, mediated by surface integrins and adhesion molecules, confer a biological advantage over synthetic carriers. That said, protein corona formation in biological fluids can compromise the targeting performance of synthetic nanoparticles. On the other hand, EVs face scalability and batch-to-batch consistency challenges that affect the reproducibility of their homing properties (9).

Scalability, batch reproducibility, and manufacturing complexity are major hurdles for nanomedicine clinical translation. Lipid nanoparticles have the most established scalability and reliable reproducibility, with several approved products on the market (84, 86). Even so, conventional batch methods still show batch-to-batch variability. Polymeric nanoparticles have seen more limited clinical progress. Traditional methods like emulsification and nanoprecipitation often cause shifts in particle size and drug loading during scale-up. Newer technologies such as microfluidics and high-pressure homogenization show promise, but their solvent compatibility, process robustness, and cost-effectiveness still need further validation (85, 86). Microfluidics has notably improved size and dispersity control for both lipid and polymeric platforms, though widespread adoption remains constrained by cost, throughput limitations, and compatibility with specific materials (87–89). EVs face the greatest manufacturing challenges. Their yields are intrinsically low, purification is labor-intensive, sources are heterogeneous, and standardized quality control is lacking. Although 3D culture and cell stimulation can boost output, the batch consistency and functional fidelity of their complex cargo still fall short of what synthetic carriers can achieve (84, 88, 90).

Immunological safety is also a key factor in determining clinical translatability. Lipid and polymeric nanoparticles, as synthetic materials, can influence immune responses through their surface chemistry and breakdown products, for example by modulating APCs polarization and cytokine profiles. At the same time, both carrier types carry some risk of complement activation or nonspecific uptake, which may lead to immunogenicity (9, 84). EVs are generally considered less immunogenic because they are naturally derived. However, their complex cargo including MHC molecules, miRNAs, and cytokines can mediate either immunosuppressive or pro-inflammatory effects depending on the cell source and pathological context, rendering their safety profile inherently two-sided.

## 3 Payload categories and immunomodulatory mechanisms of nanodelivery systems

### 3.1 Small-molecule immunosuppressive agents

Since 1999, RAPA has been clinically utilized to prevent graft rejection owing to its effective inhibition of T-cell activation and proliferation. It blocks mTOR signaling via FKBP12 binding to stall T cells at the G1/S transition, meanwhile boosting Foxp3^+^ Treg formation and suppressing pro-inflammatory T helper 17 (Th17) cell differentiation (91, 92). Beyond direct mTOR-dependent T cell cycle arrest, RAPA promotes TGF-β-dependent formation of alloantigen-specific induced Tregs, induces IL-10-producing Bregs, and drives DCs toward a tolerogenic phenotype with low MHC class II and CD80/CD86 expression (93–98).

Burke et al. (99) employed polyethylene glycol-b-polypropylene sulfide nanocarriers for subcutaneous RAPA delivery, which not only mitigated drug-associated adverse effects, but also induced CD4^+^ T-cell anergy through costimulatory blockade on APCs, as evidenced by downregulated CD40, CD80 and CD86 expression on DCs and monocyte and macrophage populations. This tolerogenic modulation established antigen-specific hyporesponsiveness in islet allografts, supported by mixed lymphocyte reaction (MLR) showing selective suppression against donor antigens with intact third-party reactivity, while also reducing neutrophil infiltration and promoting regulatory T cell expansion. Yet all functional data were generated in murine models, and whether this subcutaneous APC-targeting strategy can achieve similar tolerogenic effects in large animals or humans remains untested, leaving its translational relevance uncertain (100, 101).

Tacrolimus is an FDA-approved macrolide immunosuppressant (102, 103). Zeng et al. (104, 105) demonstrated that the combination of tacrolimus and mycophenolate mofetil enhanced Treg suppressive function in liver transplant recipients, increasing Treg-mediated inhibition of effector T cell proliferation and reducing IFN-γ and tumor necrosis factor-alpha (TNF-α) production. This combination upregulated coinhibitory receptors on Tregs, particularly programmed death 1 (PD-1) and TIGIT, along with cytotoxic T-lymphocyte-associated protein 4 (CTLA-4), Tim-3, and LAG-3. Concurrent blockade of PD-1 and TIGIT attenuated Treg activity and restored inflammatory cytokine secretion, confirming their role in Treg-mediated immunoregulation. Furthermore, mPEG-PLGA-PLL nanocarriers co-delivering tacrolimus and anti-IL-21R antibody selectively targeted secondary lymphoid organs (106), suppressed T follicular helper cells proliferation and B-cell activation, reduced IgG production, mitigated tacrolimus-related nephrotoxicity and systemic inflammation, and decreased intragraft lymphocyte infiltration, thereby attenuating acute rejection and promoting graft survival in transplanted tissues. Nevertheless, skin transplant rejection mechanisms differ from vascularized solid organ transplants, and the complex antibody conjugation process raises concerns about batch-to-batch consistency and long-term stability (107, 108).

### 3.2 Nucleic acid payloads

As a key costimulatory pathway, blockade of the CD40-CD40L axis effectively inhibits T-cell activation and promotes Treg expansion, positioning it as a potential strategy for inducing immune tolerance. It suppresses APC-derived inflammatory cytokines without affecting surface molecules, interrupts CD4^+^ T-cell help that sustains CD8^+^ effector differentiation, and drives plasmacytoid DC-dependent conversion of CD4^+^ Foxp3^-^ T cells into iTreg (109–111). Wang et al. (112) engineered PEG-PLGA nanoparticles for the delivery of CD40 small interfering RNA (siRNA), distinct from anti-CD40 antibodies, which not only down-regulated CD40 expression on mature DCs and macrophages, but also targeted hematopoietic stem cells and myeloid progenitors to inhibit their differentiation into functional APCs, thereby suppressing APCs maturation, reducing proinflammatory cytokine secretion, and impairing alloreactive T-cell priming, ultimately prolonging skin allograft survival in murine. Still, siRNA delivery efficiency in large animals remains uncertain, and the multi-dose intravenous regimen raises questions about clinical feasibility. Another study utilized PEG-b-PLGA nanoparticles to co-deliver Cas9 mRNA and CD40-targeting guide RNA, enabling precise CD40 knockout in DCs at the genomic level, which reduced CD40 expression and concomitantly downregulated CD80 and CD86 on DCs. This consequently suppressed T-cell activation, elevated the Treg proportion, alleviated graft injury, and prolonged graft survival. Yet, *in vivo* editing efficiency remained limited to approximately 11% and graft prolongation was partial rather than complete tolerance, while off-target effects and long-term safety of CRISPR remain unresolved (113).

Beyond siRNA and mRNA, microRNA and long non-coding RNA have also been exploited for tolerance induction (114). Li et al. (115) constructed a mesenchymal stem cell-derived exosome delivery platform that co-delivered a DC-SIGN aptamer/mTOR siRNA chimera and regulatory miRNAs to target DCs. By inhibiting the DC-SIGN/mTOR signaling pathway and sustainably upregulating HLA-G expression, this platform prolonged allograft survival during the 14-day observation period and promoted HLA-G mediated immune regulation. However, the short observation window did not confirm long-term tolerance maintenance, and the multi-step preparation process raises scalability concerns. Wu et al. (116) reported that long non-coding RNA DANCR, carried by bone marrow-derived mesenchymal stem cell exosomes, downregulated SIRT1 expression in CD4^+^ T cells, by directly binding to SIRT1 and promoting SMURF2-mediated ubiquitination and degradation of SIRT1, which in turn upregulated Foxp3 and IL-10 expression and promoted Treg differentiation, thereby alleviating acute rejection in kidney transplantation during the 6-day observation period. Similarly to the exosome strategy above, both studies had short observation periods, and the large-scale production and quality control of exosome preparations remain unresolved.

### 3.3 Immunomodulatory proteins

The PD-1/PD-L1 and CTLA-4/CD80 axes are central immune checkpoint pathways governing T-cell activation and peripheral immune tolerance. PD-L1 has been widely investigated in tumor immunology (117, 118), and its role in transplant tolerance is increasingly being explored (119). Liao et al. (120) reported that Toll-like receptor (TLR) stimulation can generate TLR-activated Bregs, it activated Bregs express high levels of PD-L1 and induce PD-1 expression in T cells, thereby promoting Treg generation and prolonging allograft survival. Mechanistically, PD-L1 on TLR-Bregs is required for optimal induction of Foxp3^+^ Tregs with enhanced functional markers, including CCR7 to promote Treg migration and CTLA-4 to reinforce suppressive capacity, along with IL-10 production. By contrast, PD-L1-deficient TLR-Bregs fail to induce these functional Tregs or prolong graft survival. Whether this PD-L1-dependent mechanism is replicable in human Bregs remains to be determined. Building on this PD-L1-mediated tolerogenic, Ou et al. (121) further demonstrated that PD-L1-modified small EVs markedly prolong graft survival by enhancing Foxp3^+^ CD25^+^ Treg differentiation from naive CD4^+^ T cells through PD-1/PD-L1 signaling, upregulating anti-inflammatory cytokines including IL-2, IFN-γ, TGF-β, and IL-10, while dose-dependently inhibiting CD4^+^ T-cell proliferation and reducing Th17 and Th1 effector responses, ultimately promoting immune tolerance in vascularized composite allografts. Nonetheless, the multi-step manufacturing process raises scalability concerns, and the short observation window precludes confirmation of long-term tolerance maintenance.

In addition to the PD-1/PD-L1 axis, the CTLA-4/CD80 pathway represents another key target for modulating alloimmune responses (122). For instance, dual-targeting exosomal nanovesicles directed against PD-L1 and CTLA-4 effectively extend the survival of skin and cardiac allografts by engaging PD-1 on T cells and CD80 on DCs to co-inhibit T cell activation and proliferation, reducing CD8^+^ T cell infiltration and inflammatory cytokine production including TNF-α, IL-6 and Granzyme B, while promoting Foxp3^+^ CD25^+^ Treg cell expansion and TGF-β1 secretion, thereby establishing a more tolerogenic immune microenvironment. Even so, the complex cell engineering and multi-step preparation raise scalability concerns, and all validation remains in murine models (123).

Indoleamine 2,3-dioxygenase (IDO), predominantly expressed in lymphoid tissues and the placenta, mediates the immunoregulatory functions of Tregs (124). Notably, crosstalk between IDO and Tregs plays an indispensable role in CTLA4-Ig-induced cardiac allograft tolerance in murine (125). He et al. (126) demonstrated that exosomes derived from IDO1-overexpressing rat BM-MSC prolonged short-term graft survival by downregulating CD40, CD86, CD80 and MHC class II on DCs while upregulating PD-L1, expanding Tregs, reducing CD8^+^ T cells, suppressing pro-inflammatory cytokines (IL-1, IL-1β, IL-2, IFN-γ, IL-18) and elevating anti-inflammatory mediators (IL-10, TGF-β1/β2/β3). Mechanistically, these exosomes carried elevated FHL-1 protein, upregulated miR-540-3p and downregulated miR-338-5p, targeting JAK3 and RAG2 respectively to reinforce tolerance. Nevertheless, the observation window was limited to 7 days, precluding confirmation of long-term tolerance maintenance, and the multi-step process involving IDO1 overexpression and exosome isolation raises scalability concerns.

### 3.4 Cytokines

The immunomodulatory cytokine TGF-β1 orchestrates diverse immune processes and serves a critical role in establishing peripheral T-cell immune tolerance (127, 128). However, the *in vivo* application of soluble TGF-β1 is severely limited by its short half-life (129), off-target effects (130), and the pro-fibrotic risks associated with high-dose administration (131). Li et al. (132) fabricated PLGA microspheres for localized, controlled release of TGF-β1, which efficiently promoted the differentiation of naive CD4^+^ T cells into Foxp3^+^ CD25^+^ iTregs in a dose-dependent manner, with conversion efficiencies comparable to soluble TGF-β1, and these iTregs exhibited robust suppressive function equivalent to natural Tregs in both polyclonal and antigen-specific settings. Although monotherapy failed to significantly prolong graft survival, cotransplantation with allogeneic islets confirmed the feasibility of local immunomodulatory biomaterial-based delivery systems.

Horwitz et al. (133) further engineered CD2 antibody-decorated PLGA nanoparticles co-encapsulating TGF-β and IL-2, generating tolerogenic nanoparticles that expanded CD4^+^ and CD8^+^ Treg subsets and induced donor-specific hyporesponsiveness, as evidenced by reduced MLR to donor alloantigen while preserving third-party reactivity. Additionally, these nanoparticles promoted tolerogenic DCs characterized by downregulated MHC class II, CD80 and CD86. However, the study was validated only in splenocyte transfer models assessing MLR suppression and cell persistence, without evaluation of graft survival in solid organ transplant models, and did not compare the nanoparticle treatment to low-dose IL-2 or TGF-β monotherapy controls.

Hepatocyte growth factor represents another cytokine with potent immunomodulatory activity (134–136). Chen et al. (137) demonstrated that hepatocyte growth factor suppresses acute alloreactivity and prolongs liver allograft survival through a dual mechanism. It directly induces apoptosis of recipient allostimulated CD8^+^ T cells via cMet-mediated Fas signaling pathway activation, while indirectly promoting regulatory T-cell differentiation by driving monocytes into regulatory DC-like cells that produce IL-10, upregulate ILT3 and IDO, and reduce costimulation. Nevertheless, the pharmacokinetic limitations of hepatocyte growth factor and lack of large-animal validation place this approach at the mechanistic exploration and proof-of-concept stage (134).

### 3.5 Donor-specific antigen

Induction of antigen-specific immune tolerance with donor antigens constitutes a major research focus in the field of transplantation (138). Numerous donor antigen delivery strategies have been developed, and their combination with immunomodulators significantly enhances tolerance induction efficacy. Bryant et al. (41) utilized PLGA nanoparticles covalently conjugated with donor splenocyte lysate proteins for delivery to recipient splenic APCs, including macrophages, B cells, and DCs. This formulation targeted the indirect allorecognition pathway by driving clonal expansion followed by contraction and deletion of indirectly activated CD4^+^ T cells. Monotherapy achieved 20% tolerance, while coadministration with a short course of low-dose RAPA synergistically elevated the rate to approximately 60%. Even so, the undefined nature of whole cell lysate and inability to target direct-pathway T cells cap its efficacy, placing it at the proof-of-concept delivery optimization stage. Immature DCs constitute a natural source of donor antigens, imDex maintain the native conformation of donor MHC antigens and act as functional donor antigen complexes.

Li et al. (139) demonstrated that imDex, which express low levels of MHC class I molecules and costimulatory molecules CD80/CD86 yet retain moderate MHC class II molecules, synergized with a subtherapeutic short course of RAPA, an mTOR inhibitor that blocks DCs maturation and expands Tregs, to suppress recipient T-cell proliferation and activation against donor antigens while increasing splenic CD4^+^ CD25^+^ FoxP3^+^ Tregs, thereby inducing donor-specific tolerance (MST = 92 days, 3/6 >100 days), as confirmed by second graft acceptance (>100 days vs. Third-party rejection) and adoptive transfer of tolerance (splenocytes: MST = 83 days), whereas monotherapies were ineffective. Nonetheless, this strategy involves complex donor DCs culture and exosome isolation, raising clinical feasibility concerns.

Likewise, combined administration of imDex and Tregs elicited immune tolerance in a rat liver transplantation model (6/9 >100 days) with donor-specific MLR unresponsiveness, mechanistically attributed to imDex-mediated expansion of Tregs in a recipient DC-dependent manner. However, the strategy involves dual cell culture processes, including imDex isolation and 14-day Treg expansion, limiting clinical feasibility, and its efficacy without immunosuppression in large animals or humans remains unknown (140). Furthermore, model antigens have been utilized to mimic donor antigens for delineating tolerance induction mechanisms. Wang et al. (141) administered the model antigen ovalbumin (OVA) orally prior to murine skin transplantation, which promoted the generation and expansion of OVA-reactive Tregs and rendered CD4^+^ and CD8^+^ T cells anergic, as evidenced by upregulated CD73 and FR4 expression, impaired cytokine production upon restimulation, and reduced systemic accumulation of antigen-reactive T cells, thereby ultimately establishing donor-specific immune tolerance to OVA-expressing skin grafts. Yet efficacy with MHC alloantigens was modest, and reliance on a tolerogenic environment in gut-draining lymph nodes that is vulnerable to infection raises clinical controllability concerns.

### 3.6 Combination payloads

Compared with single-payload immunomodulatory strategies, combination payloads facilitate more robust induction of transplant tolerance through synergistic targeting of multiple immune pathways. As a core immune checkpoint axis (142), the PD-1/PD-L1 pathway is routinely combined with immunosuppressants to construct synergistic delivery platforms. Yang et al. (143) engineered PD-L1 nanovesicles encapsulating RAPA to synergistically enhance immunosuppression and prolongskin allograft survival. Specifically, surface-displayed PD-L1 on the nanovesicles engaged PD-1 receptors on T cells to activate the PD-1/PD-L1 coinhibitory axis, while encapsulated RAPA inhibited AKT/mTOR signaling, leading to reduced S6 and AKT phosphorylation and subsequent suppression of T-cell activation and proliferation. Moreover, co-delivery reduced the effective RAPA dosage and significantly mitigated its adverse side effects. Similarly, dual modulation via PD-L1 and RAPA effectively suppressed transplant immune responses and promoted the establishment of a tolerogenic microenvironment. Even so, the platform involves complex cell engineering, membrane vesicle isolation, and electroporation-based drug loading, and validation remains limited to a murine skin transplant model (144).

Luo et al. (145) utilized DC-derived exosomes overexpressing PD-L1 and harboring donor antigens in a cardiac transplantation model. These exosomes concurrently presented donor antigens and PD-L1 coinhibitory signals to H2^b^-reactive CD4^+^ and CD8^+^ T cells either directly or semi-directly via cross-dressing with recipient DCs, thereby delivering dual signals to donor-specific T cells. This dual presentation substantially prolonging antigen-specific graft survival by suppressing T-cell activation, inducing apoptosis of activated T cells via the mitochondrial pathway (upregulated Bax/Bcl-2 ratio and cleaved caspase-3), and promoting their differentiation into Tregs (increased splenic CD25^+^ Foxp3^+^ Treg proportion, elevated TGF-β1 and IL-10, and reduced IFN-γ and TNF-α). Nevertheless, the approach relies on lentiviral transduction of a DC line and exosome isolation, and achieved only prolonged survival without true tolerance.

Furthermore, co-delivery of antigens and immunomodulators via nanocarriers constitutes a highly effective strategy. Kim et al. (146) co-encapsulated OVA and dexamethasone within PLGA nanoparticles, which preferentially targeted phagocytes, mitigated the systemic side effects of dexamethasone, and elicited the generation of tolerogenic DCs characterized by a low CD86 high PD-L1 phenotype, reduced pro-inflammatory cytokines, and elevated IL-10. These tolerogenic DCs subsequently drove the differentiation of naive T cells into FOXP3^+^ Tregs, thereby establishing OVA-specific immune tolerance. Yet the non-specific immunosuppressive effects of dexamethasone may obscure the true contribution of antigen-specific tolerance, and graft survival has not been evaluated in transplant models.

The studies discussed above illustrate a broad spectrum of nanomaterial-based delivery strategies, which differ considerably in their choice of carrier materials, encapsulated payloads, and experimental transplantation models. To provide a clear overview, we summarize the key features of these representative studies in **Table 1**, including nanocarrier type, particle size, administration route, combination therapy, transplant model, species, toxicity, graft survival duration, follow-up period, donor-specific tolerance evidence, and major limitations.

**Table 1:** Summary of nanomaterial carriers, encapsulated payloads, and therapeutic outcomes in various allogeneic transplantation models.

Nanomaterial carriers	Payload types	Nanoparticle size	Administration route	Combination therapy	Transplantation models (solid-organ/tissue/cellular)	Species	Toxicity	Duration of graft survival (MST)	Follow-up duration	Donor-specific evidence	Major limitations	References
PLGA	Antigen,Anti-Fas mAb,TGF-β,PD-L1-Fc,CD47-Fc	202.4nm	i.v.	–	Skin (Tissue)	Murine	No significant toxicity	61 d	45 d	H-2K^b^-reactive CD8^+^ T cell reduced by 90.3%, no effect on third-party (BALB/c)	Single MHC mismatch, no rechallenge or adoptive transfer	(38)
PLGA	Donor antigen peptides	486.5 ± 26.4 nm	i.v.	–	Skin (Tissue)	Murine	No obvious chemical toxicity	36 d (20-35% survival to 90 d)	90 d	third-party antigen stimulation eliminates graft protection	Partial protection only, no rechallenge, single MHC II molecules mismatch	(40)
PLGA	Donor antigen	458.8 ± 14.8 nm	i.v.	RAPA	Islet (Cellular)	Murine	Not directly assessed	PLG-dAg: ~20% survival to 100 d, PLG-dAg +RAPA: ~60% survival to 100 d	100 d	third-party SJL/J antigen conferred no protection in BALB/c islet grafts	Indirect pathway only, direct pathway unaffected, no rechallenge	(41)
Chitosan/PLA	RAPA	312–326 nm	Topical	–	Corneal (Tissue)	Rabbit	No obvious ocular toxicity	27.2 d (50% survival to 45 d)	45 d	–	Corneal model (immune-privileged site), no third-party or rechallenge assays.	(52)
Donor-derived imDex	MHC	40–90 nm	i.v.	LF 15-0195	Heart (Solid-organ)	Rat	No significant toxicity	>200 d (100% survival at 200 d)	200 d	Second donor-matched grafts accepted (>200 d) while third-party rejected	Tolerogenic non-T regulatory cells unidentified	(68)
Treg cell-derived exosomes	–	30–100 nm	i.v.	–	Kidney (Solid-organ)	Rat	Not assessed	21 d	21 d	Donor-type Treg exosomes superior to autologous and B-cell exosomes	Exosomal cargo molecules (miRNA/protein) not identified, short follow-up	(69)
Treg cell-derived exosomes	–	~100 nm	i.v.	–	Liver (Solid-organ)	Rat	Not assessed	90 d	90 d	–	Exosomal cargo undefined, donor specificity unverified	(70)
imDex	–	30–150 nm	i.v.	–	Kidney (Solid-organ)	Murine	Not assessed	16 d	20 d	–	Donor specificity unverified, single murine renal model only	(210)
PEG-PLGA	FK506	85 ± 10 nm	i.v.	–	Liver (Solid-organ)	Rat	No significant toxicity	> 60 d	60 d	–	Rat single model, no donor-specific tolerance validation	(76)
PEG-b-PPS	RAPA	~100 nm	s.c.	–	Islet (Cellular)	Murine	Low toxicity	83% recipients maintain normoglycemia over 100 d	100 d	Donor-specific MLR hyporesponsiveness (donor Balb/c inhibited vs. Third-party C3H intact)	No secondary graft rechallenge, lacking large-animal validation	(99)
mPEG-PLGA-PLL	Anti-IL-21R mAb + FK506	248.1 nm	i.v.	–	Skin (Tissue)	Murine	No significant toxicity	Grafts survived to 7 d	7 d	–	Donor-specific tolerance unverified, only skin graft model without vascular organ validation	(107)
PEG-PLGA	CD40SiRNA	~100 nm	i.v.	–	Skin (Tissue)	Murine	No significant toxicity	>18 d	~20d	MLR suppressed donor and third-party responses	Non-specific pan-suppression rather than true donor-specific tolerance	(112)
PEG-b-PLGA	Cas9 mRNA+guide CD40 RNA	~100 nm	i.v.	–	Skin (Tissue)	Murine	No significant toxicity	18 d	20 d	–	Donor-specific tolerance not validated, indirect allorecognition incompletely suppressed	(113)
MSC-derived exosomes	DC-SIGN/mTOR chimera	~20–100 nm	i.v.	–	Skin (Tissue)	Murine	Not assessed	14 d	14 d	–	Donor specificity unverified, no *in vivo* third-party controls	(115)
BM-MSC exosomes	lncRNA DANCR	~100 nm	i.v.	–	Kidney (Solid-organ)	Murine	Not assessed	Graft survival assessed at day 6 only	6 d	–	Donor specificity unverified, short follow-up precludes long-term tolerance assessment	(116)
PD-L1-modified BM-MSC EVs	–	40–200 nm	i.v.	RAPA	VCA (Tisssue)	Murine	Not assessed	Prolonged survival to 39.4 ± 2.4 d	40 d	–	Donor specificity unverified, lacking free PD-L1 comparative controls	(121)
Cellular exosome-like nanovesicles	PD-L1+CTLA-4	~180 nm	i.v.	–	Skin (Tissue) and Heart (Solid-organ)	Murine	No significant toxicity	both graft survival to ~9 d	14 d	–	Donor specificity unverified, no free PD-L1/CTLA-4 protein or Ig controls to validate NV delivery superiority	(123)
IDO1-overexpressing rat BM-MSC-derived exosomes	–	–	i.v.	–	Heart (Solid-organ)	Rat	Not assessed	Only 7-day observation	7 d	–	Donor specificity unverified, no long-term graft survival data	(126)
PLGA	TGF-β1	~54 µm	Local co-transplant	–	Islet (Cellular)	Murine	No significant toxicity	25% survival > 90 d	90 d	MLR showed intact responses to both donor and third-party antigens	No systemic donor-specific tolerance,TGF-β1 MP monotherapy failed to significantly prolong graft survival	(132)
CD2 Ab-modified PLGA	IL-2+ TGF-β	268.5 ± 12.7 nm	i.p.	–	Allogeneic lymphocyte adoptive transfer (Cellular)	Murine	Not assessed	Only 30-day immune follow-up	30 d	Donor-specific MLR hyporesponsiveness confirmed with Treg expansion and tolerogenic DCs	Donor specificity indicated by MLR but unverified *in vivo*, splenocyte model only, lacking vascularized organ validation	(133)
imDex	–	~50–100 nm	i.v.	RAPA	Heart (Solid-organ)	Murine	Not assessed	92 d	100 d	Second donor graft accepted (>100 d vs. third-party 21 d); splenocytes adoptively transferred tolerance (MST = 83d); donor-specific MLR inhibition	Non-T regulatory cell mechanism undefined, Treg donor specificity unverified, no large-animal validation	(139)
imDex	–	~30–120 nm	i.v.	Donor-specific Tregs	Liver (Solid-organ)	Rat	Not assessed	>100 d	100 d	Donor-specific MLR inhibition; antigen-specific Treg function *in vitro*	imDex-DC antigen presentation axis undefined, only rat liver model, lacking translational validation.	(140)
PD-L1 nanovesicles from HEK293T cell membranes	RAPA	170–180 nm	i.v.	–	Skin (Tissue)	Murine	No significant toxicity	Prolonged survival ~ 9d	14 d	–	No rechallenge/third-party assays confirming donor-specific tolerance,mechanism of PD-L1/RAPA synergy not fully elucidated	(143)
PD-L1-Overexpressing exosomes	Donor antigen	90–200 nm	i.v.	–	Heart (Solid-organ)	Murine	No significant toxicity	11d	30 d	Donor-specific graft prolongation (B6 prolonged vs. C3H not) and MLR inhibition	No rechallenge tests to confirm durable donor-specific tolerance	(145)

PLGA, poly(lactic-co-glycolic acid); PLA, polylactic acid; TGF-β, transforming growth factor-beta; PD-L1, programmed death ligand 1; CD47-Fc, CD47-Fc fusion protein; Anti-Fas mAb, anti-Fas monoclonal antibody; MHC, major histocompatibility complex; RAPA, rapamycin; PEG-b-PPS, polyethylene glycol-block-polypropylene sulfide; mPEG-PLGA-PLL, methoxypolyethylene glycol-poly(lactic-co-glycolic acid)-poly-L-lysine; FK506, tacrolimus; siRNA, small interfering RNA; MSC, mesenchymal stem cell; BM-MSC, bone marrow-derived mesenchymal stem cell; lncRNA, long non-coding RNA; VCA, vascularized composite allotransplantation; imDex, immature dendritic cell-derived exosomes; DC, dendritic cell; EV, extracellular vesicle; MST, median survival time; MLR, mixed lymphocyte reaction; IDO, indoleamine 2,3-dioxygenase; TNF-α, tumor necrosis factor-alpha; IFN-γ, interferon-gamma; IL-10, interleukin-10; HLA-G, human leukocyte antigen G; Treg, regulatory T cell; CTLA-4, cytotoxic T-lymphocyte-associated protein 4; Foxp3, forkhead box P3; mTOR, mechanistic target of rapamycin; Tfh, T follicular helper; LN, lymph node;d, day; i.v., intravenous; s.c., subcutaneous; i.p., intraperitoneal.

## 4 Core delivery strategies and *in vivo* modes of action of nanodelivery systems for transplantation tolerance induction and graft protection

### 4.1 Tolerance-inducing organ-targeted delivery

Tolerance-associated organ-targeted delivery stands as a core strategy for nanodelivery-mediated transplantation tolerance induction. Through precise targeting of key immune-regulatory organs, this strategy mitigates systemic immunosuppression and facilitates robust local immune modulation (147, 148).

Lymph nodes function as the primary site for naive T-cell activation and antigen presentation, critically dependent on the stromal scaffold sustained by fibroblastic reticular cells (FRCs). Within the lymph node, APCs, particularly DCs, process and present donor alloantigens via MHC molecules to alloreactive T cells, thus constituting a critical regulatory hub for modulating transplant alloimmunity (149–151). Zhao et al. (147) demonstrated that lymph node-resident FRCs are indispensable for anti-CD40L-mediated long-term cardiac allograft acceptance. FRCs sustain lymphoid architecture and recruit T cells via CCL19 and CCL21. Upon anti-CD40L treatment, FRCs acquire a tolerogenic phenotype that promotes Treg differentiation while suppressing effector T cells. Conversely, FRC depletion abrogates this effect by impairing T cell and DC trafficking to lymph nodes. Recent studies further demonstrate that distinct FRCs subsets serve as critical stromal regulators capable of fostering tolerogenic lymph node microenvironments. However, the complex nanoparticle fabrication and unclear targetability of human FRCs remain unresolved (152).

Accordingly, surface functionalization of nanocarriers with lymph node-homing molecules such as CCL21-specific aptamers or tannic acid achieves selective drug accumulation in draining lymph nodes. This strategy prolongs graft survival in a murine chronic cardiac transplant models while mitigating off-target uptake mediated by the mononuclear phagocyte system in liver and spleen tissues. Tannic acid-modified nanoparticles raised lymph node tacrolimus levels 15-fold and extended rat graft survival to 36 days but stopped short of permanent tolerance. Macrophage-based PD-L1 and RAPA co-delivery platforms added mechanistic depth yet remained in mice with complex manufacturing challenges (153, 154).

As an immune-privileged organ characterized by intrinsic tolerogenic properties, the liver exhibits robust innate tolerance yet remains vulnerable to sustained immune reactivity following transplantation (155). Studies have demonstrated that antigen peptide-loaded PLGA nanoparticles efficiently target the liver and promote local Tregs induction via preferential uptake by liver sinusoidal endothelial cells and Kupffer cells. Liver sinusoidal endothelial cells cross-present encapsulated antigens through MHC class II molecules to naive CD4^+^ T cells, driving FoxP3^+^ Treg differentiation while secreting IL-10 and TGF-β. Kupffer cells reinforce this tolerogenic environment through Fas/FasL-mediated T-cell apoptosis and additional IL-10 production, collectively establishing antigen-specific immune tolerance (79).

### 4.2 Immune cell-targeted delivery

Immune cells stand as the core regulatory units of the transplant immune response. Targeted delivery to immune cells enables precise modulation of transplant immunity, thereby constituting a promising approach for nanodelivery-mediated transplantation tolerance induction. DCs represent the most potent APCs *in vivo* and serve as critical targets for tolerance induction (156). DC-SIGN is a key molecular target for DC-specific targeting, upon which surface-engineered porous silicon nanoparticles can be constructed, providing a novel platform for the precise delivery of immunosuppressive agents to DCs. Recent studies have developed DC-targeted porous silicon nanoparticle systems co-loaded with C-type lectins, DC-SIGN ligands, and CD11c monoclonal antibodies, achieving highly efficient DC-selective delivery (157).

Macrophages represent the predominant immune cells infiltrating grafts during the early phases of transplantation, and their polarization toward the proinflammatory M1 phenotype is tightly linked to graft rejection (158). Vichare et al. (159) engineered a locally injectable, macrophage-targeted nanoemulgel platform for tacrolimus delivery. Upon internalized by activated macrophages, the nanoemulsion payload delivered tacrolimus intracellularly, suppressing LPS/TLR4-triggered M1-polarized macrophage activation via calcineurin and ERK/MAPK inhibition, and thereby downregulating TNF-α, IL-6, and nitric oxide production. All data were generated from *in vitro* macrophage experiments, with no *in vivo* evaluation of pharmacokinetics, graft accumulation, or anti-rejection efficacy in any transplant animal model, leaving a considerable gap to true proof-of-concept for local graft-targeted immunosuppression.

Moreover, self-assembled nanoparticles have been utilized to target macrophages and promote their polarization toward the anti-inflammatory M2 phenotype, which effectively attenuates graft rejection and extends skin graft survival. These nanoparticles preferentially accumulate in the spleen and are predominantly phagocytosed by splenic macrophages, in which they suppress TLR-4/NF-κB/NLRP3 signaling cascades, reduce TNF-α, IL-6, IL-1β, and IL-18 secretion, upregulate CD206 expression, and inhibit MHC class II molecules. This macrophage repolarization subsequently decreases IFN-γ and increases IL-4 in T cells, shifting the Th1/Th2 balance toward a tolerogenic state, though the strategy has been validated only in a skin graft model and achieves prolongation rather than permanent tolerance, leaving a considerable gap to clinical translation (160).

Beyond DCs and macrophages, regulatory subsets including Tregs and Bregs, alongside effector T cells, also constitute critical targets for immune cell-targeted delivery. The future development of cell type-specific targeting molecules against distinct immune cell subsets will enable multicellular coordinated modulation of the transplant immune response, thereby further augmenting the efficacy of transplantation tolerance induction.

### 4.3 Graft site-targeted local delivery

Local immune modulation and tolerance induction at the graft site effectively circumvent the adverse effects elicited by systemic immunosuppression (161, 162). Through the sustained and controlled release of bioactive agents such as cytokines and immunosuppressive drugs, this strategy has emerged as a potential approach to enhancing allograft survival and reducing rejection. By virtue of their distinctive physicochemical properties, nanomaterials offer essential technical support for precise local delivery, enhancing local drug concentrations at the graft site while reducing off-target effects and systemic toxicity.

Local delivery systems have exhibited promising translational potential across diverse transplant models. In corneal endothelial transplantation, nanoparticle-mediated targeted delivery significantly enhances the survival of engrafted cells and facilitates tissue repair (163, 164). In peripheral nerve allotransplantation, a nanofiber-based local delivery system mediates sustained local release of tacrolimus, thereby effectively suppressing T cell-mediated rejection (165). Moreover, hydrogels loaded with trophoblast EVs enable sustained release of tolerogenic factors at the cell transplant site, thus promoting the establishment of local immune tolerance (166).

In islet transplantation, establishing an immune-protective local microenvironment is critical for preventing graft rejection. Kuppan et al. (167) utilized PLGA microparticles to locally co-deliver cyclosporine A and cytotoxic T lymphocyte-associated antigen 4-immunoglobulin fusion protein to islet grafts. The long-term risk of local cyclosporine A toxicity to β-cells remains unaddressed, and all data are from murine models alone. Localized CsA suppressed effector T-cell proliferation via calcineurin-dependent IL-2 inhibition, while CTLA4-Ig blocked CD28-mediated costimulation. This dual strategy reduced intra-graft CD4^+^/CD8^+^ T-cell and macrophage infiltration, and downregulated proinflammatory cytokines and chemokines that drive β-cell apoptosis and rejection, successfully achieving long-term survival of murine allogeneic islet grafts and inducing donor-specific operational tolerance.

Likewise, PLGA-mediated delivery of a glucagon-like peptide-1 (GLP-1) analog (168) or GLP-1-conjugated nanofiber hydrogels (169) improves graft survival via anti-inflammatory effects and islet function preservation. Localized GLP-1 release reduces pro-inflammatory cytokines, attenuates hypoxic injury via suppression of hypoxia-related genes, and activates AKT signaling to exert anti-apoptotic effects, while GLP-1-conjugated hydrogels resist enzymatic degradation and sustain bioactivity at the transplant site, collectively promoting islet engraftment and preserving β-cell function (170, 171).

Local delivery to the graft site allows for sustained, high-concentration drug release at the target site, thereby directly remodeling the local immune microenvironment. This strategy represents a powerful approach to mitigating systemic side effects and improving the efficiency of transplantation tolerance induction. Highly amenable to both solid organ and cell transplantation, this modality holds substantial promise for future clinical translation.

**Figure 1:**
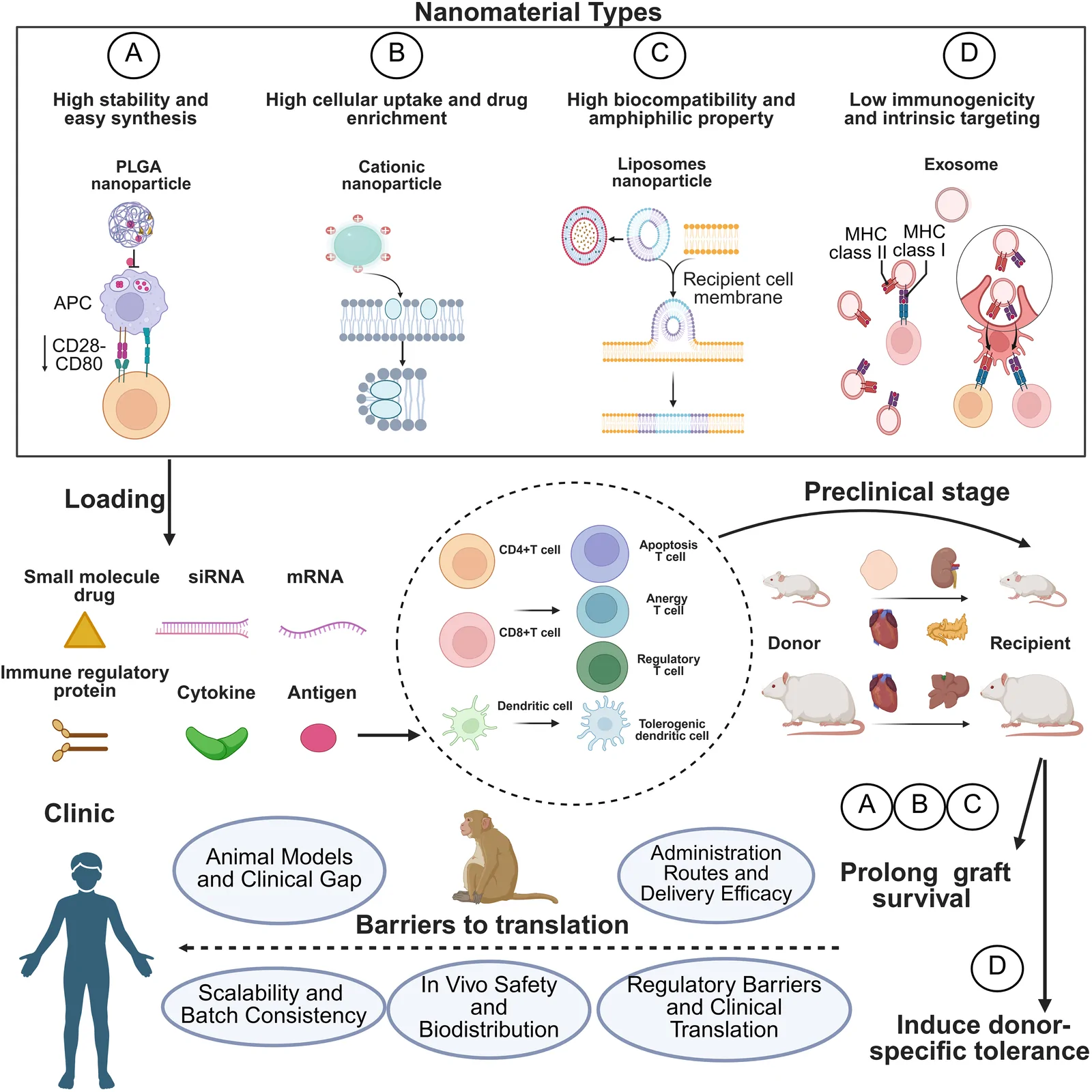
Nanocarriers for transplant tolerance: mechanisms and translational barriers. This schematic depicts the nanocarrier platforms used for transplant tolerance induction, their immunomodulatory mechanisms, and the barriers to clinical translation. (1) Nanomaterial types: including PLGA nanoparticles **(A)**, liposomes **(B)**, cationic nanoparticles **(C)**, and exosomes **(D)**, offering advantages such as high stability, enhanced cellular uptake, high biocompatibility, and low immunogenicity. (2) Cargo payloads: nanoparticles can deliver small molecule drugs, siRNA, mRNA, immune regulatory proteins, cytokines, and antigens. (3) Immunomodulatory mechanisms: nanoparticles modulate antigen-presenting cells and T-cell fate to induce T-cell apoptosis, anergy, or regulatory T cell differentiation, thereby establishing donor-specific tolerance or prolonging graft survival. (4) Barriers to translation: including the animal model to clinical gap, scalability and batch consistency, *in vivo* safety and biodistribution, administration routes and delivery efficacy, and regulatory and clinical translation hurdles. Created with BioRender.com.

Nanocarrier-based strategies for transplant tolerance are fully illustrated in **Figure 1**. This schematic detail four key sections: various nanomaterial platforms, diverse therapeutic cargos, immune-regulatory pathways driving tolerance, and major clinical translation obstacles, offering a holistic view of the preclinical advances and translational constraints of these delivery systems.

### 4.4 Nanomaterial-based strategies for pre-transplant organ preservation and ischemia-reperfusion injury mitigation

Organ preservation and ischemia-reperfusion injury are two interconnected challenges that arise before transplantation yet have a lasting impact on long-term graft outcomes. Static cold storage at approximately 4°C remains the clinical standard, but prolonged cold ischemia on its own causes Adenosine Triphosphate (ATP) depletion, mitochondrial dysfunction, and reactive oxygen species (ROS) accumulation (172). The abrupt restoration of oxygen upon reperfusion does not simply reverse these changes. Instead, it amplifies oxidative stress, activates innate immunity, and triggers a cascade of inflammatory responses (173). These pathological events not only compromise graft viability directly but also increase its immunogenicity, making the graft more susceptible to subsequent rejection.

To address these challenges, recent studies have explored nanomaterial-based interventions from various angles. The strategies covered in this review fall into three broad categories.

One approach delivers energy substrates directly to ischemic cells. Atchison et al. (174) used PR_b-functionalized liposomes to deliver ATP to ischemic β cells. The PR_b peptide binds α_5_β_1_ integrin to facilitate liposome internalization, and the co-delivered ATP and lipids together maintain metabolic activity and suppress necrosis after ischemic injury. Grignano et al. (172) took a different carrier strategy, loading ATP into mesenchymal stem cell-derived EVs via microfluidic technology. Their ATP-EVs exhibited better ATP retention than liposomes in the extracellular environment and more effectively restored intracellular ATP levels while reducing apoptosis in ischemic renal tubular epithelial cells.

Another strategy aims to scavenge ROS while modulating inflammation. Dong et al. (175) constructed platelet membrane-coated PLGA nanoparticles co-encapsulating quercetin and rapamycin. The platelet membrane proteins ICAM-1 and P-selectin enable targeting to injured vascular endothelium and activated macrophages, respectively. Quercetin scavenges ROS via the Nrf-2/HO-1 pathway, while rapamycin suppresses JNK signaling in M1 macrophages to attenuate inflammatory injury. In a rat liver transplantation model, this dual-agent sequential strategy significantly reduced ALT and AST levels and alleviated biliary injury.

A third category exploits the intrinsic properties of extracellular vesicles for multi-target protection. Tolomeo et al. (176) evaluated mesenchymal stem cell-EVs in a porcine donation after circulatory death heart transplantation model. EV-treated hearts showed better-preserved mitochondrial cristae, delayed release of cardiac enzymes, and fewer caspase-3-positive cells. These positive outcomes in a large-animal model lend greater translational credibility than data from murine studies alone.

Collectively, these nanomaterial strategies, which range from energy delivery and ROS scavenging to inflammation modulation and EV engineering, offer viable intervention pathways for organ preservation and ischemia-reperfusion injury mitigation (177). These pre-transplant approaches not only improve immediate graft function but also reduce allograft immunogenicity, thereby creating conditions more favorable for subsequent tolerance induction.

## 5 Challenges and translational status of nanodelivery systems in transplantation tolerance induction

### 5.1 Inherent biological limitations of nanomaterials

Biological limitations of nanomaterials pose significant challenges to their clinical translation, primarily relating to biosafety, manufacturing reproducibility, and *in vivo* metabolic behavior. The degradation kinetics of certain synthetic polymeric nanomaterials *in vivo* are difficult to regulate accurately. Excessive local accumulation of degradation products may induce mild chronic inflammation or tissue damage (178, 179), thereby compromising the establishment of a tolerogenic immune microenvironment at the graft site. Natural polymeric nanomaterials exhibit substantial batch-to-batch heterogeneity in physicochemical properties and broad molecular weight distribution, leading to inconsistent drug loading efficiency, targeting specificity, and *in vivo* therapeutic efficacy, thereby markedly hindering standardized industrial production (180, 181).

Endogenous nanoscale delivery vehicles such as EVs are limited by high production costs, low yields, and immature surface engineering techniques. These drawbacks impede efficient loading of drugs or nucleic acids and preclude large-scale clinical translation (182). Furthermore, most nanomaterials have a short systemic circulation half-life and are rapidly cleared by the mononuclear phagocyte system (183, 184), resulting in insufficient accumulation at target sites and inadequate immune modulation (185).

### 5.2 Interference from the *in vivo* immune microenvironment

The immune microenvironment following allogeneic transplantation exhibits remarkable complexity and dynamics. Post-transplant, recipients undergo a cascade of pathophysiological events, including inflammatory activation, immune cell infiltration, and cytokine dysregulation. These events directly impair the *in vivo* targeting efficiency, drug release kinetics, and therapeutic efficacy of nanodelivery systems. Specifically, the acute inflammatory milieu at the graft site elicits neutrophil activation, thereby accelerating phagocytic clearance of nanoparticles and drastically reducing drug delivery to target cells (186). Moreover, memory T cells confer innate resistance to nanomaterial-mediated immunosuppressive regimens (187). Even if such systems effectively abrogate the activation and clonal expansion of naïve effector T cells, memory T cells retain the capacity for rapid proliferation and elicit delayed-type graft rejection, constituting a formidable barrier to robust transplantation tolerance induction (188).

Importantly, the long-term evolution of the immune microenvironment leads to instability in achieving persistent tolerance. Most current nanodelivery platforms only prolong short-term graft survival and fail to induce stable, long-term donor-specific tolerance. The tolerogenic effects of several approaches gradually diminish over time, increasing the susceptibility to delayed chronic rejection. On one hand, nanodelivery systems are incapable of sustaining durable immunomodulation. Late-stage alterations in the immune microenvironment frequently trigger phenotypic instability of induced Tregs, leading to the loss of their immunosuppressive function (189). On the other hand, insufficient targeted modulation of chronic rejection-associated pathologies-including vascular endothelial injury and interstitial fibrosis-further compromises the establishment of long-term tolerance (190).

### 5.3 Technical barriers and quality control imperatives in clinical translation

Despite encouraging preclinical results, the clinical translation of nanodelivery platforms for transplantation tolerance remains confronted by formidable hurdles spanning manufacturing, regulatory, and biological domains.

Scalable good manufacturing practice-compliant manufacturing with consistent batch-to-batch reproducibility remains a critical translational bottleneck (191). The multi-step production process is highly sensitive to minor variations in parameters such as homogenization speed, agitation time, reagent concentration, freezing rate, solvent mixing ratio, and sterilization conditions, which can significantly alter critical quality attributes including particle size, surface charge, morphology, and drug loading. Physicochemical properties including particle size, surface charge, and drug-loading efficiency strongly influence *in vivo* performance, yet large-scale production fails to ensure high batch-to-batch uniformity (192–194). Moreover, the lack of validated standardized characterization methods hampers the detection of subtle batch differences (195, 196).

Ensuring sterility of nanomedicines presents another major translational challenge. Available terminal sterilization methods such as autoclaving, irradiation, and ethylene oxide treatment can compromise nanoparticle integrity, drug stability, or biological activity, while sterile filtration is often limited by particle size and recovery yield. Endotoxin removal is further complicated by nanoparticle-assay interference and surface adsorption (197).

Once administered, nanoparticle clinical translation is critically challenged by rapid clearance mediated by the mononuclear phagocyte system, including Kupffer cells in the liver and macrophages in the spleen, which recognize and engulf opsonized nanoparticles through phagocytosis. This process, governed by particle size, surface charge, and PEGylation, leads to substantial off-target accumulation in the liver, spleen, and lungs, drastically reducing bioavailability at the intended site (198, 199). The route of administration is a critical factor. Systemic delivery (oral or intravenous) leads to widespread distribution and toxicity, whereas local administration can achieve higher local concentrations with fewer systemic side effects. However, the clinical translation of these localized approaches remains limited by interspecies pharmacokinetic differences, manufacturing complexity, and the lack of standardized regulatory frameworks for locally delivered nanomedicines (200).

Nanoparticle toxicity and immunogenicity remain major translational barriers. Physicochemical properties such as size, surface charge, and coating can induce oxidative stress, inflammation, and complement activation. Endotoxin contamination, which affects over 30% of nanoformulations, frequently interferes with safety assays and produces false-positive or false-negative immunotoxicity results. Furthermore, the lack of standardized detection methods to differentiate material-specific effects from endotoxin interference, together with poor predictability of animal models for human immune responses, severely hampers clinical translation (200–202).

Most nanodelivery systems in preclinical research are evaluated in small animal models. However, marked differences in physiological architecture and immune systems between rodents and humans impede accurate prediction of pharmacokinetics, pharmacodynamics, and biosafety in humans, creating substantial interspecies translation barriers (203). For example, lymph node-targeting strategies effective in small animals often exhibit drastically reduced efficiency in humans due to divergent lymphatic circulatory networks. Murine are bred in specific pathogen-free environments with predominantly naive immune systems and lack constitutive MHC class II molecules expression on vascular endothelium, making it significantly easier to induce tolerance in murine than in humans who possess abundant alloreactive memory T cells.

Moreover, most murine transplantation models are homogeneous acute models using sub-therapeutic immunosuppression, whereas human rejection is chronic and heterogeneous, evolving over extended periods despite immunosuppression. Large animal models such as pigs and nonhuman primates offer greater anatomical and physiological resemblance to humans, yet their widespread use is hindered by limited research reagents, challenges in transgenic manipulation, high costs, and lack of institutional infrastructure. Notably, Lipid nanoparticle-mediated mRNA delivery to CD34^+^ cells in rhesus monkeys has been achieved without mobilization or conditioning, suggesting that nanoparticle delivery in nonhuman primates is feasible at a proof-of-concept level (204). Similarly, localized delivery of FOXP3-engineered Treg exosomes via a hydrogel system demonstrated preliminary feasibility in a porcine model, suggesting that exosome-hydrogel combination strategies may offer a translatable approach for local immune tolerance induction in renal transplantation (54). Nonetheless, moving from such preliminary demonstrations to rigorously validating nanoparticle-based tolerance strategies under clinically relevant conditions remains a substantial gap (200).

In addition, high production costs represent a widespread limitation, particularly for EVs and highly modified polymeric nanomaterials, which precludes cost-effective large-scale clinical application (205, 206). The economic burden is further magnified by the relatively small market size for transplantation compared to oncology or autoimmune diseases, reducing the financial incentive for industry to invest in the development and regulatory approval of novel nanodelivery platforms for tolerance induction (200).

Current FDA and EMA guidelines lack a unified definition of nanomaterials and fail to provide formulation-specific, standardized characterization requirements, leaving critical quality attributes such as particle size, surface chemistry, and drug release to be evaluated on a case-by-case basis without harmonized protocols (196, 207). Meanwhile, multi-component delivery systems lack standardized quality control for drug content, release kinetics, and production safety, and no clinically compliant evaluation system has been established (208, 209).

Hybrid new drug application pathways can reference existing safety and efficacy data, offering a promising regulatory route for nanotechnology-based products. This is feasible only when the nanomaterial demonstrates meaningful clinical improvement, such as enhanced bioavailability, reduced dosing frequency, or active targeting. Nevertheless, the lack of stringent acceptance criteria and the difficulty in establishing bioequivalence for complex generics continue to hinder commercialization. This underscores the urgent need for convergence among academia, industry, and regulatory agencies to establish robust, product-specific quality standards and scalable manufacturing protocols (207). To date, no nanodelivery platform specifically designed for transplantation tolerance induction has entered clinical testing; the field remains entirely preclinical, highlighting the critical need for standardized characterization methods, reliable large-animal studies, and clearer regulatory guidance to bridge the translational gap.

### 5.4 Clinical translation landscape and registered trials

Despite the extensive preclinical evidence accumulated over recent decades, the translation of nanomaterial-based platforms into clinical testing in transplantation remains unexpectedly sparse. A search of the ClinicalTrials.gov registry as of July 2026 identifies only a handful of registered trials that explicitly involve nanotechnology in the context of solid organ transplantation, and the majority remain in early-phase exploration. The key features of these representative trials are summarized in **Table 2**.

**Table 2:** Clinical translation landscape of nanomaterial-based platforms in transplantation.

Nanoparticle type	Therapeutic cargo	Transplantation setting	Clinical phase	Main objectives	NCT number
LNP	HBV antigen-encoding mRNA	Liver transplantation (HCC)	Phase I	Safety and preliminary efficacy of LNP-mRNA therapeutic vaccine in liver transplant recipients with HCC	NCT07077356
EVs	EV-derived proteins (diagnostic biomarkers)	Heart transplantation	Observational	EV-derived protein profiling for early diagnosis of acute cardiac allograft rejection	NCT07673536
I5NP	p53-targeting siRNA	Kidney transplantation (DGF prevention)	Phase I/II	Safety and clinical activity of I5NP in preventing delayed graft function	NCT00802347

LNP, Lipid nanoparticles; HBV, Hepatitis B virus; HCC, Hepatocellular carcinoma; EVs, Extracellular vesicles; siRNA, small interfering RNA; DGF, delayed graft function.

Among these, the types of nanoplatforms and their intended applications are far from uniform. The LNP-mRNA vaccine (NCT07077356) exemplifies a therapeutic strategy in which nanotechnology serves as an active immunotherapy vehicle, in this case to prevent hepatocellular carcinoma recurrence after liver transplantation. In a distinctly different vein, mesenchymal stem cell-derived extracellular vesicles have entered clinical testing as a liquid biopsy tool for non-invasive monitoring of cardiac allograft rejection via EV protein profiling (NCT07673536). A third category involves nanoparticles as direct interventional agents against peri-transplant ischemia-reperfusion injury. The siRNA-based candidate I5NP (NCT00802347), which targets the p53 pathway to reduce tubular epithelial cell apoptosis in kidney transplantation, stands as one of the few examples in this direction.

Notably, no registered clinical trial to date has been identified with the direct goal of inducing transplantation tolerance as its primary endpoint, a sobering reminder of the gap between the extensive preclinical literature on tolerance induction and the near-absence of clinical validation.

## 6 Conclusions and discussions

In this review, we have organized four nanocarrier platforms, five payload categories, and three delivery strategies into a single coherent framework. Unlike previous reviews that address biomaterials, nanomedicine, and transplant immunity separately, we offer an integrated mechanistic analysis and a unified system for comparing different carriers. We also take a close look at key translational barriers, from material biology and production issues to the limitations of current animal models, and map out possible solutions. Overall, this review provides a broad, cross-disciplinary perspective that has not been offered in earlier publications.

Despite the progress summarized above, the translation of nanodelivery systems for transplantation tolerance remains at an early stage. Current challenges include insufficient *in vivo* stability and targeting precision of nanocarriers, the lack of standardized manufacturing and quality control protocols, and the scarcity of predictive large-animal models that adequately reflect human transplant immunology. Moreover, whether the tolerance induced by existing strategies can be maintained over the long term has not been rigorously evaluated beyond short-term observation windows.

Future efforts should therefore focus on several specific areas. Carrier design needs to be refined through advanced materials engineering to improve circulation stability, tissue selectivity, and controlled release kinetics. The mechanistic basis of nanocarrier-immune cell interactions should be systematically investigated using integrated multi-omics and high-resolution imaging approaches to guide rational design. Standardized quality control criteria and scalable production processes must be established to meet regulatory requirements for clinical translation. Preclinical validation should be extended to large-animal models with extended follow-up to assess the persistence and safety of induced tolerance. In addition, combination strategies that integrate nanodelivery systems with established tolerance-inducing regimens, such as chimeric antigen receptor-based approaches or gene editing, warrant systematic exploration to enhance therapeutic consistency and efficacy. Addressing these issues will be critical to determining whether nanodelivery-based tolerance induction can ultimately benefit transplant recipients.

### 6.1 Literature search strategy

We searched PubMed and Web of Science (1990-2026) using Boolean operators with keywords divided into three categories: transplantation, nanomaterials, and immunity. Within each category, terms were combined with OR, and the categories were linked with AND. This narrative review was designed for thematic coverage and mechanistic interpretation, not for systematic quantitative synthesis. After removing duplicates via NoteExpress, we conducted screening in two stages. We first evaluated titles and abstracts, giving priority to *in vivo* allotransplant studies as primary evidence while retaining *in vitro* or non-transplant studies only for mechanistic reference. We then assessed full texts and selected articles based on novelty, experimental quality, and thematic consistency. We applied uniform exclusion criteria, removing conference abstracts, patents, and studies outside transplantation or nanomaterial immunomodulation. We also manually scanned reference lists of included papers and key reviews to capture additional reports. The initial search produced hundreds of records, and after screening we chose over 200 representative articles for detailed discussion.
